# Supplementary figures and images for: ROM1 is redundant to PRPH2 as a molecular building block of photoreceptor disc rims
Source: eLife. 2023 Nov 22;12:RP89444. doi: 10.7554/eLife.89444 (PMC10665016; doi:10.7554/eLife.89444)

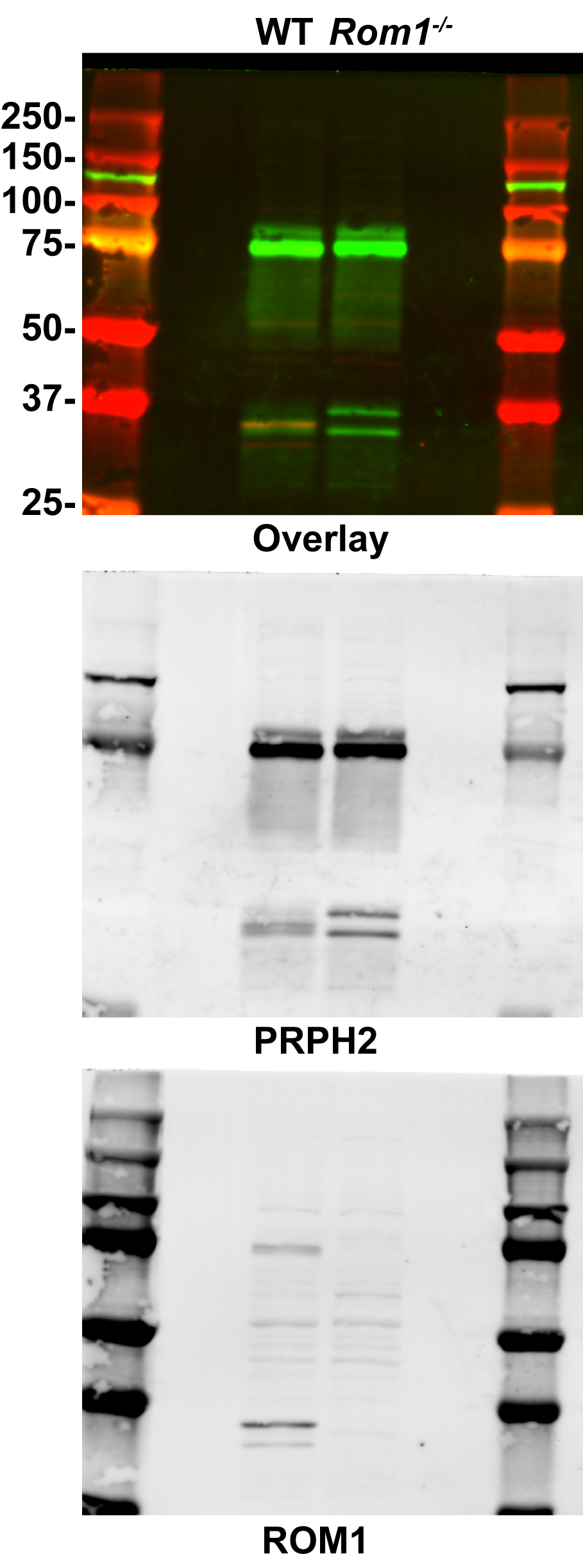

Supplement: Figure 5—source data 1. [file elife-89444-fig5-data1.zip › Figure 5 - source data 1.tif]

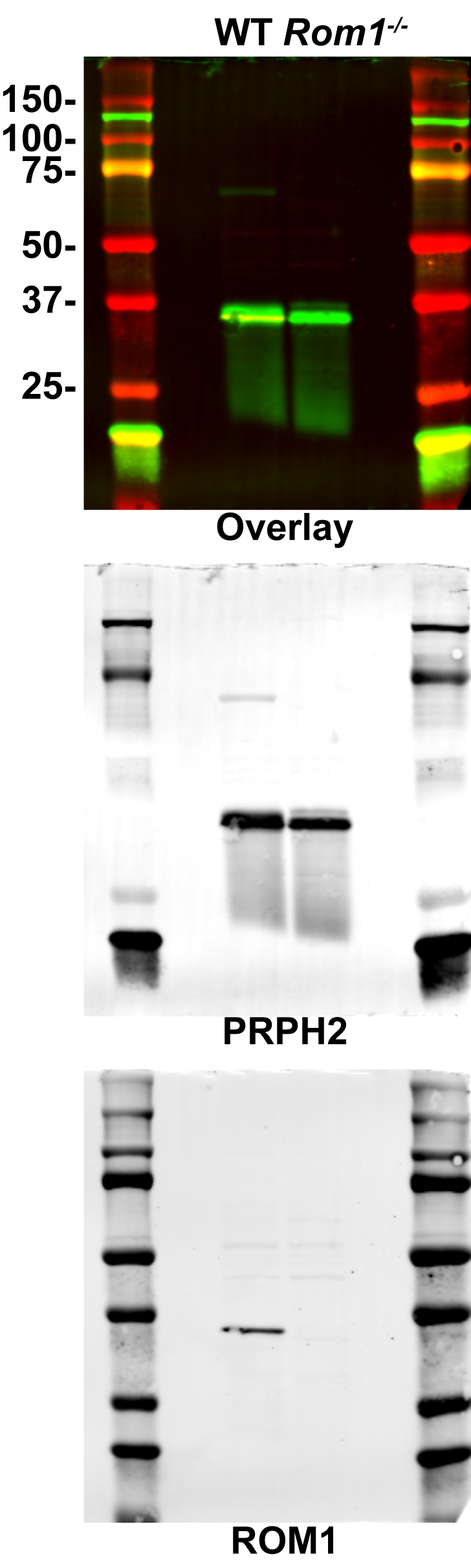

Supplement: Figure 5—source data 2. [file elife-89444-fig5-data2.zip › Figure 5 - source data 2.tif]

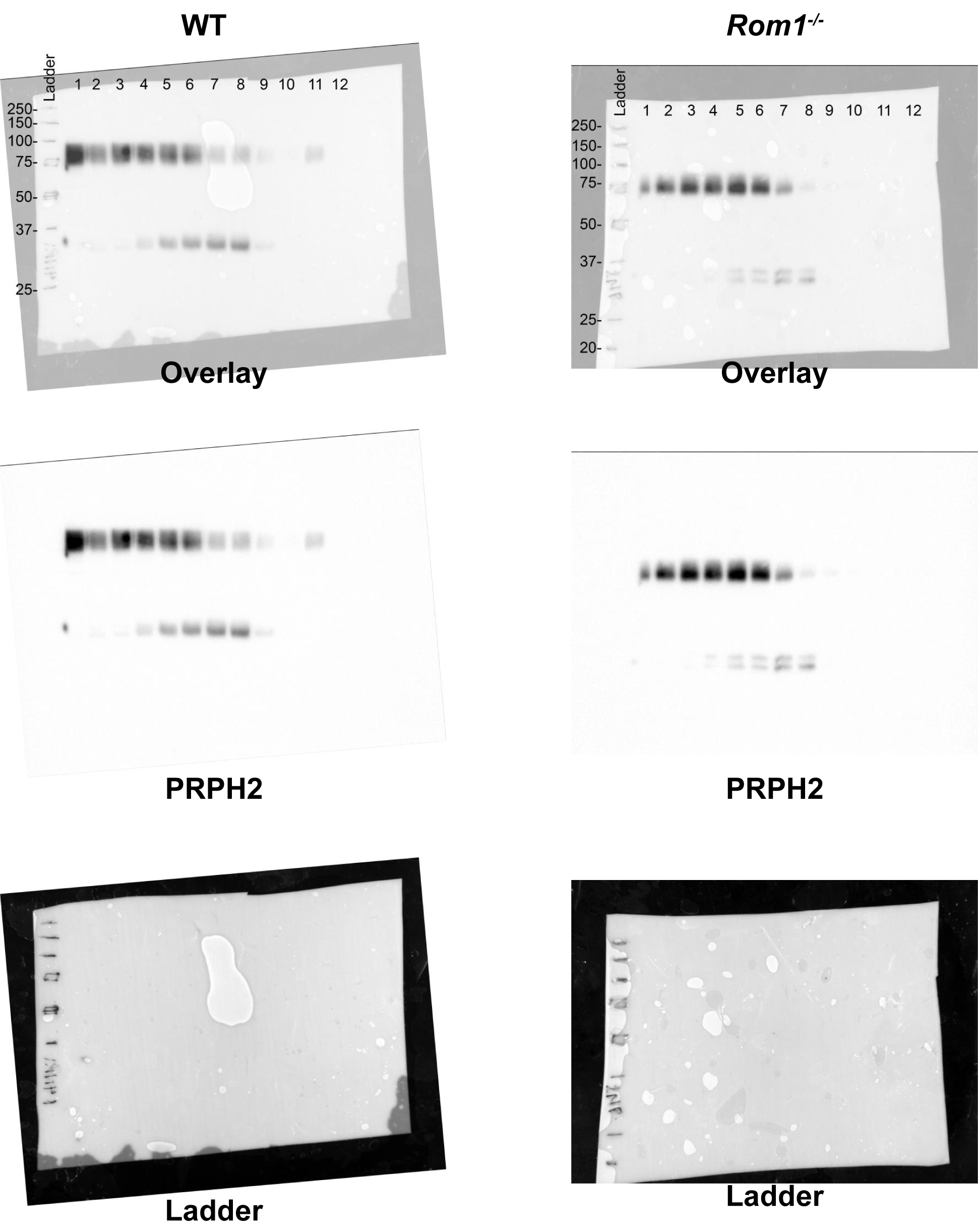

Supplement: Figure 5—source data 3. [file elife-89444-fig5-data3.zip › Figure 5 - source data 3.tif]

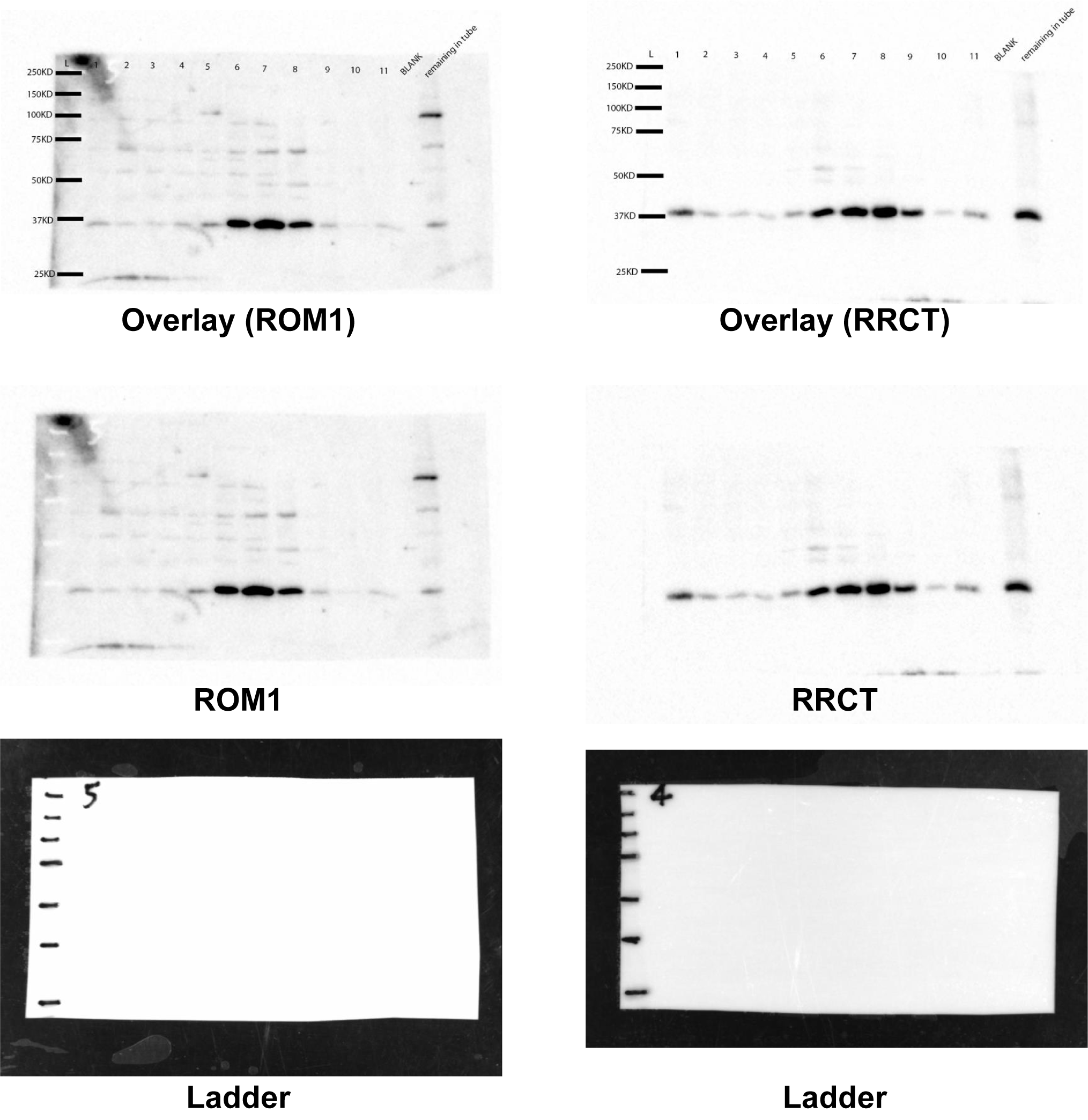

Supplement: Figure 6—source data 1. [file elife-89444-fig6-data1.zip › Figure 6 - source data 1.tif]
